# Central auditory processing and interhemispheric integration: the relationship between speech sound coding disorder and articulation disorder

**DOI:** 10.1038/s41598-026-56590-7

**Published:** 2026-06-09

**Authors:** Işık Sibel Küçükünal, Fulya Yalçınkaya

**Affiliations:** 1 Faculty of Health Sciences, Department of Language and Speech Therapy, Gazi University, 06490 Ankara, Türkiye; 2 Professor Emeritus, Sinop, Türkiye

**Keywords:** Neuroscience, Psychology, Psychology

## Abstract

This study aims to demonstrate that speech sound disorders associated with central auditory processing disorder stem from a processing disorder in the central auditory pathways during listening, rather than from an articulation-based disorder. Included 25 children with articulation problems (WAP; 7 females, 18 males) and 25 typically developing children without articulation problems (WoAP; 10 females, 15 males), aged 5–8 years. Assessments comprised the Hacettepe Articulation Test, Scan-C subtests (Filtered Words, Auditory Figure-Ground, Competing Words, Competing Sentences), and the Denver II Developmental Screening Test. The Children in the WAP group scored low on the left ear only in the dichotic words and dichotic sentences tests. Left ear performance was significantly lower in the dichotic words test (*p* = 0.026, Cohen’s d = 0.609) and the dichotic sentences (*p* = 0.001, r = 0.427). These results suggest that articulation disorders may indicate a central auditory processing deficit, particularly a delay in right-hemisphere auditory maturation. It has been observed that in children with central auditory processing disorder, whatever speech sounds they process defectively during listening, they also produce those same sounds defectively. In this context, this study offers a new perspective on the relationship between articulation disorders and central auditory processing. Therefore, it is recommended that central auditory processing screening be conducted in children with articulation disorders to distinguish the underlying disorder.

## Introduction

Speech Sound Disorders (SSD) is a common communication impairment characterized by persistent difficulties in producing speech sounds, which can reduce intelligibility and impact verbal communication. It may also co-occur with language impairments, complicating diagnosis and intervention^1,2^. Approximately 3.6% of children aged 4–8 years experience SSD, with over 76,000 annual referrals to NHS Speech and Language Therapy services, underscoring its public health importance^1^. Understanding the emergence of SSD requires considering the auditory processes involved in articulation. Accurate articulation depends on the correct neural encoding and decoding of speech sound frequencies within the central auditory pathways^3,4^. Postnatal maturation of auditory nerve pathways through myelination and synaptogenesis provides the physiological basis for Central Auditory Processing (CAP) development^5^. CAP enables individuals to distinguish speech sounds from environmental noise, thereby facilitating speech comprehension. Sounds of varying frequency, intensity, duration, and speed are transmitted through the auditory system—including the inner ear, auditory nerve, brainstem nuclei, and auditory cortex—where target speech sounds are identified and linked to specific words and conversations^6^. Investigating the relationship between articulation disorders and CAP is critical not only for diagnostic accuracy but also for shaping intervention approaches. Auditory processing supports accurate phonemic representation, speech perception in noise, and the development of accurate articulation and literacy skills. Contemporary evidence emphasizes that Central Auditory Processing Disorder (CAPD) can co-occur with articulation/speech-sound disorders and may contribute to persistent articulation difficulties even when peripheral hearing is normal^7–10^. For this reason, routine CAP assessment in children with articulation disorders will facilitate both accurate diagnostic classification and targeted therapeutic planning.

The term CAPD is an explanation of a condition in which an individual has no hearing impairment but experiences difficulty processing auditory information^11^. CAPD prevalence in school-age children is commonly reported as 2–7%, depending on diagnostic criteria, test battery, and the population studied^9,12,13^. The literature consistently indicates notable co-occurrence between central auditory processing deficits and speech production problems. However, precise prevalence figures are heterogeneous and depend strongly on diagnostic criteria, CAPD batteries, SSD subtypes, and study populations. The strongest direct prevalence figure within articulation disorders is from Alqudah et al.'s study, with Musiek-based CAPD assessment reporting 72.5% APD in a sample of 51 articulation-disordered children^14^.

The relationship between CAPD and SSD is both complex and significant. Neurological abnormalities in auditory pathways may underlie both conditions^15,16^. The neurophysiological relationship between CAPD and SSD is based on the dynamic interaction between the processing of auditory stimuli at cortical and subcortical levels and speech motor output. The acoustic properties of speech sounds (frequency, duration, intensity, temporal variations) are encoded in higher-order auditory cortical areas such as the superior temporal gyrus (STG)^17^. The encoded acoustic information is transferred via the arcuate fasciculus and the corpus callosum between Broca’s area (speech production) in the left hemisphere and the right hemisphere’s areas that process prosodic and suprasegmental features^18,19^. The right ear typically shows an advantage during dichotic listening due to direct transmission to the language-dominant left hemisphere^20^. Studies of individuals with CAPD indicate a significant reduction in performance in the left ear, particularly during dichotic listening tests^21,22^. This finding suggests that auditory information processed in the right hemisphere is not adequately transmitted to the language areas in the left hemisphere via the corpus callosum^6,20^. Furthermore, it has been shown that deficits in rapid auditory processing skills hinder the accurate discrimination of speech sounds, leading to articulation errors^23^. Deficits in CAP skills can negatively affect language and articulation skills^14,23^*.* These findings support the existence of shared neurophysiological mechanisms between CAPD and SSD. Delays in auditory feedback processing can impair language comprehension and sound articulation, demonstrating their direct interaction^16,23^.

SSD is commonly classified into two main types: articulation and phonological disorders. Articulation disorders involve difficulty producing specific speech sounds, while phonological disorders reflect broader challenges in understanding and applying a language’s sound system^2^.

The speaker’s voice reaches the child’s ears, initiating auditory processing. Accurate transmission of speech sound properties is essential for proper articulation, as distorted perception leads to incorrect processing. Impaired articulation may result from faulty coding and processing of speech sounds within the auditory pathway^17^.

This study examines the relationship between articulation disorders in children with typical language development who produce particular speech sounds inconsistently and their central auditory processing skills. The disorder involves temporary articulation errors under unclear auditory conditions (e.g., noise, fast speech), while speech remains normal with clear input. It refers to an articulation disorder resulting from the discrepancy between accurate and inaccurate auditory processing. Therefore, existing classifications of speech sound or articulation disorders in the literature do not encompass the group examined in this study. For example, Rvachew et al. attribute articulation disorders to factors such as cognitive, linguistic, and motor planning deficits, including apraxia^24^. Thus, the group examined here represents a distinct profile, diverging from the traditional definitions of articulation disorder or SSD in the literature. The relationship between CAPD and SSD has been widely explored in the literature. In studies, CAPD in children with SSD is associated with more persistent phonological processes and reduced consonant accuracy^7,10^ Moreover, it is emphasized that temporal processing and rapid auditory processing deficits are central to the CAPD–SSD interaction^2^. Additionally, its emphasis on the fact that CAPD is embedded in broader neurodevelopmental profiles, and that comorbid conditions can shape SSD presentation and response to CAPD-targeted interventions, underscores the need for holistic assessment and multidisciplinary management^8^. However, few studies have specifically investigated CAPD in relation to articulation disorder. This gap highlights the need for a precise classification of articulation disorder as a first step. Accordingly, the following classification has been proposed. Articulation involves physically forming speech sounds using the breath, vocal cords, tongue, lips, and teeth to create vocal expression. Drawing on this fundamental function, four distinct types of articulation disorders can be identified.

Group I: *Organic Articulation Disorder:* This disorder is unrelated to hearing or auditory processing and stems from motor coordination difficulties involving structures such as the tongue, jaw, and lips.

Group II: *Articulation Disorder Due to Delayed Language Development:* The acquisition of speech sounds is delayed in parallel with language development; this disorder typically resolves spontaneously once language development is complete.

Group III: *Persistent Articulation Disorder:* Although language development is normal, the child consistently mispronounces certain speech sounds.

Group IV: *Auditory Processing-Related Articulation Disorder (APRAD):* Language development is normal; however, the child may not process certain sounds clearly enough when listening to speech in their environment. This situation causes the same sound to be produced correctly sometimes and incorrectly at other times.

This classification introduces a new perspective to articulation disorder research by defining Group IV, which is the focus of this study’s examination of its relationship with CAP in children.

## Materials and methods

### Participants

The participants’ selection criteria (aged 5–8 years with no hearing loss, no additional neurological or psychiatric disorder diagnosis, none of the participants had a history of speech delay in early childhood, native language is Turkish, monolingual and typical development) were verified through the following means: a review of medical and educational records obtained from the relevant departments of Gazi University and the special education center affiliated with the Ankara Provincial Directorate of National Education was confirmed through family interviews.

The age range of 5–8 years was selected for several reasons. First, central auditory processing skills accelerate between the ages of 5 and 8, particularly with myelination of the corpus callosum and the maturation of interhemispheric connections^25^. This development of white matter during early childhood is directly linked to general cognitive ability^26^. Dichotic listening and speech-in-noise abilities develop substantially during this period^6^. Second, the Scan-C test battery has been standardized and validated for the 5–8 year age range in the Turkish population^27^. Third, the Hacettepe Articulation Test (HAT) norms indicate that acquisition of all Turkish consonants is typically completed by age 8^28^; including younger children would risk confounding articulation errors due to developmental immaturity rather than disorder, while including older children would increase the likelihood of compensatory strategies masking underlying deficits. The pre-school and early school years are a critical period during which children start school and acquire fundamental academic skills. In this context, the investigation of auditory processing skills in children with SSD^2^ and the identification of appropriate assessment tools for these children^1^ are of great importance. Therefore, the 5–8 year age range was chosen to optimize the validity and reliability of both auditory processing and articulation assessments while capturing a developmentally meaningful window for intervention.

The participants’ hearing, neurological, and psychiatric conditions were assessed based on existing screening records and case files. All children’s newborn hearing screening results and primary school entry hearing screening results conducted by the Ministry of National Education were within normal limits. The presence of any neurological or psychiatric diagnoses was assessed from hospital records, school files, and family interviews; children with such diagnoses were excluded from the study.

In our study, the integrity of the participants’ oral anatomy was assessed visually and functionally by an experienced speech and language therapist as part of a routine clinical assessment protocol. Children with cleft palate, macroglossia, ankyloglossia, cleft lip and palate anomalies, or any other structural anomalies were excluded from the study. It was confirmed, based on parental reports and medical records, that the participants had not previously received articulation therapy.

The children’s general developmental levels were assessed using the Denver II Developmental Screening Test^29^, and all developmental areas were found to be within age-appropriate ranges.

The participants’ language skills were assessed using the ‘language’ subscale of the Denver II Developmental Screening Test. All participants demonstrated age-appropriate performance in this area. Furthermore, it was confirmed that the participants had not received language or speech therapy support in their school records; interviews with their parents and class teachers indicated that their verbal communication skills were consistent with those of their peers.

Participants’ hand preference was assessed based on observation and parental reports. In the WAP group, 20 children (80%) were right-handed, and 5 children (20%) were left-handed. In the WoAP group, 21 children (81%) were right-handed, and 4 children (19%) were left-handed. It was concluded that hand preference did not contribute significantly to the findings in the current sample.

Participants were divided into two groups: With Articulation Problems (WAP) group (18 boys, seven girls; n = 25) and Without Articulation Problems (WoAP) group (15 boys, 10 girls; n = 25).

### Evaluation of auditory processing tests

In the Scan-C test battery, developed and standardized by Keith (2000), all stimuli are presented at a fixed intensity (50 dB SL or comfortable listening level) in the form of pre-recorded compact discs (CDs)^30^. The Turkish study was conducted by Yalçınkaya et al. on Turkish children aged 5–8 years^27^. In the Turkish version of the test, the word and sentence stimuli have been adapted to conform to Turkish phonotactic and grammatical features. In accordance with the test procedure, it is sufficient for the device used to present the stimuli (a CD player and headphones) to comply with the manufacturer’s standards; there is no need for additional calibration prior to each test in routine clinical practice. In this study, in accordance with the test procedure, stimuli were presented to all participants at the same intensity level via a high-quality laptop and Philips headphones. In our study, standard guidelines were strictly followed; prior to each subtest, participants were given verbal instructions appropriate to the purpose of the test: for example, for the Competing Words test, they were instructed as follows: 'You will hear different words in both ears at the same time; please repeat the words you hear’. Scoring was performed by calculating the correct responses for each ear, according to the test manual’s criteria.

#### Filtered words (FW)

This test presents 30 low-pass filtered monosyllabic words to each ear. The stimuli are filtered at 1000 Hz with a 32 dB roll-off per octave. The words are delivered as weak or distorted signals; this allows the test to measure the ability to understand speech in acoustically poor environments. The child is asked to repeat the words they hear. Failure to repeat indicates difficulty understanding distorted or incomplete speech stimuli and may indicate a delay in receptive language development.

#### Auditory figure ground (AFG)

Thirty words are presented to each ear against a background noise of murmurs produced by eight speakers (+ 8 dB S/N ratio). The child is asked to repeat the words they hear. Poor repetition performance may indicate a delay in the development of the auditory system.

#### Competing words (CW)

Twenty-five pairs of unrelated monosyllabic words are presented simultaneously to both ears. The child is asked to repeat the words they hear; responses are recorded according to the ear to which the words belong. The test assesses the dominant hemisphere in language and interhemispheric processing. Poor performance may indicate a delay in the maturation of the auditory nervous system or a processing disorder due to damage to the auditory pathway.

#### Competing sentences (CS)

Ten items containing three to four unrelated words are presented to each ear. The words are presented first to the right ear, then to the left ear. The child is asked to repeat the words they hear; responses are recorded according to the ear to which they belong. The test evaluates the dominant hemisphere of the language and interhemispheric processing. The results provide information about the level of maturation of the auditory nervous system and are interpreted in comparison with the CW test^27^.

### Hacettepe articulation test

The Hacettepe Articulation Test (HAT) was standardized by Yalçınkaya et al. on a sample of 753 Turkish children, and its Cronbach’s alpha reliability coefficient was found to be 0.843^28^*.* HAT is a 40-item test that assesses the production of the 21 consonant sounds in Turkish in initial and final positions within monosyllabic words. The test is conducted in a quiet environment, with the child and the examiner sitting side by side. The child was instructed to repeat the words they heard after the examiner, with the guidance: 'I am going to say some words to you now. Please repeat the words you hear after me.' To ensure the child understood, some words were spoken, and the child was asked to repeat them. Correct production of the target sound in the target position was scored as 1 point, whilst incorrect production was scored as 0 points. The total score was calculated as a percentage. Children scoring 90% or below were classified as the ‘Group WAP’, whilst those scoring above 90% were classified as the’ Group WoAP’. A certified speech-language therapist administered the tests.

### Denver II developmental screening test

The Denver II Developmental Screening Test is a screening tool designed to assess the developmental levels of children aged 0–6 years in the areas of personal-social, fine motor, language and gross motor skills. The test was standardized for use in Türkiye by Yalaz et al.^29^. During the study, the test items were presented to the child in an age-appropriate manner.

### Ethics statement

This study was conducted in accordance with the principles outlined in the Declaration of Helsinki. Ethical approval for the study was obtained from the Gazi University Ethics Committee (2025 – 1114 code number). Informed consent was obtained from a parent and/or legal guardian for all minor participants in the study.

### Statistical analysis

Data were analyzed using IBM SPSS 25. Normality was assessed via the Shapiro–Wilk test, skewness-kurtosis, coefficient of variation, and graphical methods. Mean, standard deviation, and percentage were reported for normally distributed data; median, min–max, and interquartile range were used for non-normally distributed data. Between-group comparisons were conducted using the independent samples t-test for normally distributed data and the Mann–Whitney U test for non-normal data. T-scores and Z-scores were reported accordingly. Statistical significance was set at *p* < 0.05 with a 95% confidence interval.

Effect sizes were calculated using Cohen’s d for t-tests and Pearson’s r for Mann–Whitney U tests. For Cohen’s d, values ≤ 0.2 were considered small, > 0.2–0.5 medium, and ≥ 0.8 large; for Pearson’s r, ≤ 0.1 indicated small, > 0.1–0.5 medium, and ≥ 0.5 large effects.

## Results

Examining the gender distribution of the WAP (7 females, 18 males) and WoAP (10 females, 15 males) groups, no significant difference was observed between the groups, χ^2^(1, N = 50) = 0.36, *p* = 0.55. The Chi-Square test result is not significant.

Examining the age distributions of the groups, the Shapiro–Wilk normality test results were significant for both groups (*p* < 0.001), thus the assumption of normal distribution was not met. Therefore, the non-parametric Mann–Whitney U test was used. No significant differences were found between the WAP (mean age = 74.16 ± 5.50 months, min–max = 65.00- 86.00) and WoAP (mean age = 74.56 ± 6.18 months, min–max = 62.00–87.00) groups (Mann–Whitney U = 245.50, *p* = 0.196). These findings indicate that the WAP and WoAP groups are similar in terms of demographic characteristics.

The results of the intergroup comparison using the HAT test are shown in Table 1 below.

**Table 1:** Intergroup comparison of initial syllable and final syllable in the HAT test.

Tests	With articulation problem group		Without articulation problem group				
	Mean ± SD	Min–Max	Mean ± SD	Min–Max	T Score	*p* Value	Effect Size
HAT-Initial	76.44 ± 11.14	44.44 – 94.44	94.44 ± 0.00	94.44–94.44	8.238	< 0.001	2.445
HAT-Final	76.00 ± 11.19	44.44 – 94.44	94.44 ± 0.00	94.44–94.44	8.238	< 0.001	2.494

HAT-Initial: Hacettepe Articulation Test – Initial Syllable, HAT-Final: Hacettepe Articulation Test – Final Syllable, SD: standard deviation, Min–Max: minimum–maximum.

HAT results showed that the WoAP group scored 94.44 ± 0.00 points in both syllable initial and final positions, indicating no articulation problems. In contrast, the WAP group’s results were significantly lower at both syllable initial (76.44 ± 11.14) and syllable final (76.00 ± 11.19) (*p* < 0.001). High effect sizes (Cohen’s d > 2.4) indicate that this difference is clinically and practically significant.

Table 2 below shows the accuracy and error rates at the initial and final positions for each speech sound, based on the results obtained from HAT’s WAP group. The values represent the percentage distributions of data scored as 0 (error) and 1 (correct).

**Table 2:** Speech sound error rates for HAT.

TR	IPA	Initial correct %	Initial error %	Final correct %	Final error %
b	/b/	100.0	0.0	–	–
c	/dʒ/	76.0	24.0	76.0	24.0
d	/d/	100.0	0.0	100.0	0.0
f	/f/	92.0	8.0	88.0	12.0
g	/g/	60.0	40.0	64.0	36.0
h	/h/	92.0	8.0	92.0	8.0
j	/ʒ/	60.0	40.0	48.0	52.0
k	/k/	76.0	24.0	76.0	24.0
l	/l/	64.0	36.0	72.0	28.0
m	/m/	100.0	0.0	100.0	0.0
n	/n/	100.0	0.0	100.0	0.0
p	/p/	92.0	8.0	96.0	4.0
r	/ɾ/	12.0	88.0	16.0	84.0
s	/s/	80.0	20.0	76.0	24.0
t	/t/	96.0	4.0	100.0	0.0
v	/v/	92.0	8.0	96.0	4.0
y	/j/	100.0	0.0	96.0	4.0
z	/z/	52.0	48.0	44.0	56.0
ç	/tʃ/	84.0	16.0	76.0	24.0
ğ	/ɰ/	–	–	100.0	0.0
ş	/ʃ/	72.0	28.0	68.0	32.0

The phonetic transcriptions used in the table are based on the Turkish alphabetical system. The corresponding International Phonetic Alphabet (IPA) symbols are shown. However, there are no Turkish words that begin with /ğ/ and end with /b/; therefore, the relevant cells have been left blank (indicated by "-" in the table).

The speech-based error rates reported by the WAP group in the HAT study provide insights into the nature of articulation disorders. At the onset, the highest error rates were observed in the sounds /ɾ/ (88%), /z/ (48%), /ʒ/ (40%), and /g/ (40%), whilst no errors (0%) were detected in the sounds /b/, /d/, /m/, and /n/. In the final position, the sounds with the highest error rates were /ɾ/ (84%), /z/ (56%), /ʒ/ (52%), and /ʃ/ (32%).

The fact that error rates vary widely between speech sounds, ranging from 0 to 88%, supports the existence of a spectrum of articulation disorders of varying severity within the study group. Although no direct relationship was found between the severity of articulation disorders and central auditory processing, this study identified significant differences in CAP test performance between the WAP and WoAP groups.

Although the scope of this study does not include a detailed phonetic analysis of error types (such as substitution, distortion, omission, or insertion), the findings regarding error rates provide a valuable foundation for a more detailed examination of error types in future research. In this regard, the error rates provided by the current data offer a meaningful contribution in demonstrating which sounds and positions are more prone to articulation disorders.

The findings of the Scan-C sub-tests are shown in Table 3 below.

**Table 3:** Comparison of findings from Scan-C sub-tests between groups.

Tests	With articulation problem group		Without articulation problem group				
	Mean ± SD	Min–Max	Mean ± SD	Min–Max	T Score	*p* Value	Effect Size
AFG-Left ear	68.80 ± 9.27	46.66–83.33	68.47 ± 10.04	53.33–90.00	− 0.280	0.978	− 0.007
FW-Right ear	56.00 ± 15.72	26.66–93.33	58.17 ± 14.44	20.00–80.00	0.481	0.632	0.128
FW-Left ear	51.20 ± 14.96	26.66–86.66	55.48 ± 15.90	26.66–86.66	1.029	0.308	0.275
CW-Left ear	55.20 ± 24.97	4.00–96.00	68.00 ± 17.64	32.00–100.00	2.280	0.026	0.609

AFG, auditory figure ground, FW, filtre words, CW, competing words, SD, standard deviation; Min–Max: minimum–maximum.

Competing Words—Left Ear results showed that the WAP group performed significantly worse than the WoAP group (55.20 ± 24.97 and 68.00 ± 17.64, respectively; *p* = 0.026). The moderate effect size (Cohen’s d = 0.609) proves this difference is clinically significant.

The findings of the Scan-C sub-tests are shown in Table 4 below.

**Table 4:** Comparison of findings from Scan-C sub-tests between groups.

Tests	With articulation problem group		Without articulation problem group				
	Median (IQR)	Min–Max	Median (IQR)	Min–Max	Z score	*p* Value	Effect size
AFG-Right ear	70 (16.66)	50–86.66	70 (20)	50–86.66	− 0.840	0.401	0.111
CW-Right ear	80 (36)	36–96	80 (16)	12–100	− 1.357	0.175	0.179
CS-Right ear	80 (35)	30–100	90 (30)	0–100	− 1.480	0.139	0.196
CS-Left ear	40 (30)	0–80	60 (20)	0–100	− 3.220	0.001	0.427

AFG, auditory figure ground; CW: Competing Words, Competing Sentences, Interquartile Range: IQR, Min–Max: Minimum–Maximum.

Competing Sentences Test—Left Ear (CS-Left ear) findings showed that the median value of the WAP group (40) was statistically significantly lower than that of the WoAP group (60) (*p* = 0.001). This result highlights the WoAP group’s clear superiority in left ear sentence processing skills, while supporting the WAP group’s difficulties in this area. The effect size (r = 0.427) was assessed as close to moderate, confirming the practical significance of the difference.

The AFG-Left ear, AFG-Right ear, FW-Right ear, FW-Left ear, CS-Right ear, and CW-Right ear tests in Tables 3 and 4 did not show statistically significant differences.

The tests showing significant differences were only the CW- Left ear and the CS- left ear tests.

## Discussion

This study has demonstrated a significant difference in dichotic listening tests—particularly in left-ear performance—between children with articulation disorders and those with typical development.

Children in the WAP group showed significantly lower performance on the Competing Words and Competing Sentences tests only in the left ear. Whilst there was no difference between the two groups in right-ear scores, this lower performance in left-ear scores (CW for the left ear: *p* = 0.026, d = 0.609; CS for the left ear: *p* = 0.001, r = 0.427) indicates a difficulty with interhemispheric transfer. According to Kimura’s (1961) dichotic listening paradigm, whilst the right ear transmits signals directly to the language centers in the left hemisphere, stimuli from the left ear are first processed in the right hemisphere and then transmitted to the left hemisphere via the corpus callosum^20^. Therefore, impairment in the left hemisphere generally reflects a disruption in auditory information processing from the right ear. This weakness in the transmission process causes a decrease in left ear performance in dichotic tests. Consequently, significantly lower scores in the left ear compared to the right ear indicate central auditory processing disorder^6^.

Studies in the literature involving children with speech sound disorders have similarly reported a left-ear disadvantage. In Rocha-Muniz et al.’s study of 75 children aged 6–12, groups with CAPD, language disorders, and typical development were tested using the Speech-in-Noise, Dichotic Digit, and Pitch Pattern Sequencing Tests. Left-ear scores were significantly lower than right-ear scores, and the lateralization effect in the dichotic number test was observed only in children with Specific Language Impairment^31^. Additionally, children aged 7–9 with SSD showed deficits in CAP skills, including auditory closure, figure-ground, dichotic digit, and frequency and duration pattern recognition, with a significant relationship between SSD and auditory dysfunction^7^. These findings are consistent with our study’s results.

Additionally, Vilela et al. found that a cutoff value from the Percent Consonants Correct–Revised index effectively distinguished children with CAPD from those without^32^. Furthermore, Demopoulos et al. demonstrated that rapid auditory processing of speech sounds is closely linked to articulation skills, as delays can prevent children from correctly distinguishing or accurately producing sounds^15^. It is stated that auditory processing also plays a crucial role in learning and controlling speech production, as children rely on auditory feedback to adjust syllables and speech movements according to the acoustic properties of sounds^33,34^. Children experiencing CAPD have difficulty in processing auditory information, which includes challenges in discerning speech sounds amidst background noise and following complex auditory instructions^35^. This auditory processing issue can exacerbate articulation difficulties because children may struggle to perceive and thus imitate correct sound productions, which are integral to the development of clear speech. A documented correlation exists between specific speech sounds that are consistently misarticulated and the presence of CAPD^14^.

In this context, individuals with CAPD often face challenges in speech perception, particularly in noisy environments, which can impair articulation skills^36,37^. Neural correlates of auditory processing and articulation have been studied, revealing that children with CAPD may exhibit behavioral characteristics akin to those with hearing loss, including difficulties with speech comprehension and articulation^36,38^.

The study’s most significant contribution to the literature is the identification of a previously unreported finding. The findings indicate that the decline in performance among children with articulation disorders is observed exclusively in the left ear, particularly on the Competing Words and Competing Sentences tests. That supports the concept of 'Auditory Processing-Related Articulation Disorder’ (APRAD) and suggests that its underlying mechanism may be auditory processing difficulties.

Furthermore, cognitive factors such as memory and attention further complicate the understanding of APRAD. Although traditional views often separate cognitive and auditory processing abilities in diagnosing CAPD, recent perspectives suggest a complex interplay where auditory processing inherently includes cognitive components^36,39^. That has implications for developing more effective diagnostic and therapeutic approaches for individuals affected by APRAD, highlighting the need for comprehensive assessments that include both auditory and cognitive evaluations^36,40^. Additionally, intervention studies have demonstrated that targeted auditory training and speech therapy can enhance speech outcomes for individuals with CAPD, suggesting that such disorders may benefit from neuroplastic changes facilitated by appropriate interventions^41^.

In light of these findings, traditional articulation therapies alone may not be sufficient for children with articulation disorders. In the presence of underlying auditory processing difficulties, complementary interventions such as auditory training and dichotic listening exercises may be required^42^. For this reason, it is recommended that a central auditory processing screening be included in the routine assessment of children with articulation disorders.

## Conclusions

In conclusion, this study’s findings indicate that impairments in auditory processing are reflected in speech production. When individuals process speech sounds as distorted, displaced, or omitted whilst listening, they transfer these distorted representations to production, resulting in outputs resembling articulation disorders. This study was conducted to highlight the distinction between speech sound processing disorders and production-based articulation disorders.

## Limitations

The study has certain limitations. Firstly, the relatively small sample size limits the generalisability of the findings; it is believed that studies with larger samples would yield a more robust interpretation of the results.

Furthermore, as the study included only children meeting the APRAD definition, other subgroups of articulation disorders (organic, delayed language-related, or persistent) were excluded; this presents an important area for future studies that will examine different subgroups comparatively. Whilst the study’s coverage of the 5–8 age range offers a developmental advantage, it is recommended that future studies examine the effects of age-related variables in greater detail using narrower age groups.

In addition, clinical observation was used to assess participants’ oral anatomy rather than a standard Oral Peripheral Motor Evaluation (OPME) scale; furthermore, objective audiological assessments such as pure-tone audiometry and tympanometry were not performed prior to auditory processing tests. Hearing status was based on records from the National Newborn Screening Program, the Ministry of National Education, screening results, and family reports. In future studies, it is recommended that comprehensive audiological tests be routinely administered prior to auditory processing assessments, and that oral motor assessments be conducted using standardized scales.

Additionally, because the Denver II Developmental Screening Test is standardized only for the 0–6 age range, it was used in the 7–8 age group solely as a supplementary screening tool to confirm the absence of developmental delay. However, whilst the current test provides screening information regarding general language development, it is recommended that future studies utilize comprehensive language assessment tools that evaluate receptive and expressive language skills in detail.

Finally, the use of behavioral tests in assessments has limited the examination of central auditory processing processes at the neurophysiological level. Future research involving larger samples and supported by neuroimaging methods will increase the validity and scope of the findings.

## Data Availability

The data presented in this study are available on request from the corresponding author. The data are not publicly available due to privacy and ethical restrictions.
